# Molecular epidemiology of the RD^Rio^ genotype of *Mycobacterium tuberculosis* in Ecuador: a tracking update for the years 2018 and 2023

**DOI:** 10.3389/fpubh.2026.1969751

**Published:** 2026-10-27

**Authors:** Ángel Sebastián Rodríguez-Pazmiño, Melany Patricia Cedeño-Vásquez, Liliam Paola Pozo-Estupiñán, Irina Trejo Cedeño, Greta Cárdenas Franco, Andrea Belén Salcedo Maldonado, Solón Alberto Orlando, Greta Franco-Sotomayor, Natalia Jimenez-Pizarro, Javier Hermoso de Mendoza, Miguel Angel Garcia-Bereguiain

**Affiliations:** 1 One Health Research Group, Universidad de Las Américas, Quito, Ecuador; 2 Universidad Católica de Santiago de Guayaquil, Guayaquil, Ecuador; 3 Universidad Ecotec, Samborondón, Ecuador; 4 Instituto Nacional de Salud Pública e Investigación, Guayaquil, Ecuador; 5 Departamento de Sanidad Animal, Facultad de Veterinaria, Universidad de Extremadura, Cáceres, Spain

**Keywords:** drug resistance, Ecuador, MIRU-VNTR, molecular epidemiology, *Mycobacterium tuberculosis*, RDRio genotype, whole-genome sequencing

## Abstract

**Introduction:**

The RDRio genotype of *Mycobacterium tuberculosis* (MTB), a major genotype within the Latin American-Mediterranean (LAM) family, has been associated with increased transmissibility and multidrug resistance (MDR). In Ecuador, a previous baseline study reported a low prevalence of 2.09% for 2012–2016, prior to the large influx of migrants from Venezuela—where RDRio is highly endemic—that has occurred since 2015. This study aimed to update the molecular epidemiology of the RDRio genotype in Ecuador.

**Methods:**

We screened 699 clinical MTB isolates collected in 2018 (384) and 2023 (315) for the RDRio genotype by duplex PCR and further characterized them by 24-locus MIRU-VNTR typing, spoligotyping, and whole-genome sequencing (WGS). We conducted minimum spanning tree (MST) analyses based on MIRU-VNTR profiles to assess genetic relatedness.

**Results:**

We identified 40 isolates (5.72%) as RDRio, with no significant difference in prevalence between 2018 (*n* = 20; 5.21%) and 2023 (*n* = 20; 6.35%). WGS confirmed that the dominant sublineages were 4.3.4.1 and 4.3.4.2, all within Lineage 4 (Euro-American). Genotypic resistance profiling classified 32.1% (9/28) of sequenced isolates as MDR-TB and 7.1% (2/28) as pre-XDR-TB. MST analysis showed that RDRio isolates from Ecuador segregated predominantly into distinct clonal complexes and remained largely isolated from international reference strains, including Venezuelan isolates.

**Discussion:**

The overall RDRio prevalence of 5.72% represents a nearly three-fold increase relative to the 2.09% reported for the 2012–2016 baseline, although it remained stable across the 2018–2023 period and low compared with high-endemicity settings such as Venezuela. Despite this relatively low prevalence, the RDRio genotype was associated with a substantial burden of drug resistance. The genetic segregation of the predominant RDRio Ecuadorian clonal complex from international reference strains—including Venezuelan isolates—did not support recent importation associated with the Venezuelan diaspora. Despite their limitations, classical genotyping methods remain a valid, cost-effective approach to monitor MTB strains of interest associated with MDR-TB, such as RDRio, in low-resource settings like Ecuador.

## 1 Introduction

Tuberculosis (TB), caused by *Mycobacterium tuberculosis* (MTB), remains one of the most lethal infectious diseases globally, with an estimated 10.7 million new cases and 1.23 million deaths reported in 2024 (1). The emergence and persistence of drug-resistant TB (DR-TB) strains pose a significant threat to global health control efforts, particularly in high-burden regions (2, 3). In Ecuador, TB is a critical public health concern, with the country classified as upper moderate burden, reporting an incidence rate of approximately 66.2 cases per 100,000 inhabitants in 2024. Ecuador is one of nine countries worldwide—and the only one in South America—that moved to a more severe category during 2015–2024 (1).

The population structure of MTB in Latin America is strongly dominated by the Euro-American Lineage 4, within which the Latin American-Mediterranean (LAM) sublineage is the most prevalent (4). Researchers first identified a major LAM genotype, RD^Rio^, in Rio de Janeiro, Brazil, in 2007 (5). This genotype is characterized by a unique 26.3 kb contiguous DNA deletion that results in an in-frame fusion gene, Rv3346c/55c (5). Epidemiological and clinical studies have associated RD^Rio^ strains with increased virulence, a higher frequency of cavitary pulmonary disease, and a strong tendency toward multidrug resistance (MDR) (4, 6).

In Ecuador, the first and only comprehensive baseline study of the RD^Rio^ genotype was conducted by our group using MTB isolates from 2012 to 2016 (7). That study identified a low prevalence of 2.09% and revealed a high association with drug resistance: 29.4% of the RD^Rio^ isolates were multidrug-resistant (MDR), and nearly half showed resistance to isoniazid. Since that period, the epidemiological landscape could have been reshaped by significant migratory flows, particularly from Venezuela, where the RD^Rio^ genotype is highly endemic, accounting for up to 69.8% of studied isolates (8). Since 2015, as of July 2023, at least 502,200 Venezuelan migrants have been in Ecuador (a country with a population of 17 million), a demographic shift that could have affected MTB population structure by introducing highly transmissible and resistant RD^Rio^ strains (4, 9).

This study aimed to update the molecular epidemiology of the RD^Rio^ genotype in Ecuador for 2018 and 2023 and to characterize its current prevalence, genotypic diversity and genetic relatedness, and resistance profiles.

## 2 Methodology

### 2.1 Sample collection and study design

This study analyzed 699 clinical MTB isolates collected in Ecuador during 2018 (*n* = 384) and 2023 (*n* = 315). All isolates were obtained from the strain collection of the *Laboratorio de Referencia Nacional para Micobacterias* (CRN-Micobacterias) of the *Instituto Nacional de Investigación en Salud Pública* (INSPI) in Guayaquil, the national reference center to which *M. tuberculosis* isolates from across the country are referred. This was a laboratory-based study of archived clinical isolates rather than a study recruiting participants; accordingly, the unit of analysis was the *M. tuberculosis* isolate, not the individual patient. We applied no specific inclusion or exclusion criteria beyond availability: the study population comprised all MTB isolates archived at INSPI for 2018 and 2023, originating from patients across multiple Ecuadorian provinces. All isolates came from newly diagnosed tuberculosis patients, and we included only one isolate per patient, so no patient was represented by more than one isolate within or between the two study years. The collection consisted almost entirely of pulmonary tuberculosis cases (*n* = 696; 99.57%), with only three extrapulmonary cases (0.43%), all from 2023. We classified sample type according to WHO criteria based on the site of disease; isolates without an explicit sample-type annotation in the INSPI databases were classified as pulmonary, as respiratory specimens (e.g., sputum) are frequently registered without a specified type, whereas non-respiratory specimens are consistently annotated.

We selected 2018 and 2023 as the earliest and most recent years for which cultured MTB isolates remained available and adequately preserved in the INSPI strain collection. Isolates corresponding to 2017 were no longer available owing to storage and infrastructure constraints (including recurrent power outages) affecting the collection; working under the substantial budget limitations inherent to this type of study in Ecuador, we prioritized the two extreme years of the available range (2018 and 2023) to maximize the temporal window for comparison, both between them and with the 2012–2016 baseline. For each year, we analyzed all available isolates (*n* = 384 for 2018 and *n* = 315 for 2023), corresponding to a complete census of isolates preserved at INSPI rather than a probabilistic sample. Referral and culture procedures at the national reference laboratory followed the same standardized protocols in both study years.

We performed primary isolation and culture at the INSPI CRN-Micobacterias laboratory. We grew *M. tuberculosis* by solid culture using the Kudoh–Ogawa method (10) and/or the automated MGIT liquid culture system (BD BACTEC MGIT 960), as routinely performed at INSPI. We confirmed identification of the *M. tuberculosis* complex by immunochromatographic detection of the MPT64 antigen. All procedures involving viable *M. tuberculosis*, including culture handling and harvesting, were carried out at the INSPI reference laboratory under biosafety level 2 enhanced (BSL-2+) conditions, in accordance with WHO tuberculosis laboratory biosafety guidelines, using a certified class II biological safety cabinet.

We extracted DNA by heat lysis without a commercial kit or additional extraction reagents: we harvested mycobacterial colonies, resuspended them in Tris–EDTA (TE) buffer (10 mM Tris–HCl, 1 mM EDTA, pH 8.0), and heat-inactivated them at 95 °C for 15 min. After lysis, we centrifuged samples at 10,000 × g for 5 min and used the supernatant, containing the released genomic DNA, directly as a template for molecular assays (11). We transported only heat-inactivated material to the research laboratories at Universidad de Las Américas (Quito, Ecuador), where we stored it at −20 °C until further analysis.

The study followed a stepwise genotyping approach. First, we screened all isolates for the RD^Rio^ genotype using a duplex PCR assay. We then characterized RD^Rio^-positive isolates by MIRU-VNTR genotyping and spoligotyping to assess their genetic profiles and confirm genotype. In addition, we performed WGS on a subset the RD^Rio^-positive isolates to provide high-resolution confirmation of lineage assignment and infer genotypic drug resistance.

### 2.2 Duplex PCR assays

We standardized the PCR protocol as described previously (7). We used two primer sets: one targeting a 530-bp fragment of IS1561 between the Rv3346c and Rv3355c genes as a marker for wild-type *M. tuberculosis* (WT) or a non—RD^Rio^ strain, and a second set designed to amplify a 1,175-bp region covering the RD^Rio^ locus, characteristic of the RD^Rio^ genotype. PCR reactions were performed at a final volume of 12 μL, containing 6 μL of GoTaq Green Master Mix (1X), 0.24 μL of each primer (0.2 μM), 2.4 μL of genomic DNA, and 2.64 μL of nuclease-free water. Thermocycling conditions consisted of an initial denaturation at 94 °C for 3 min; 35 cycles of 94 °C for 30 s, 61 °C for 30 s (annealing), and 72 °C for 1 min 30 s; followed by a final extension at 72 °C for 10 min. We visualized PCR products on 1.5% agarose gels run at 110 V for 2 h. We used a 100-bp DNA ladder to estimate amplicon sizes and confirm the RD^Rio^ genotype by comparing expected band lengths.

### 2.3 MIRU-VNTR PCR assays

We adapted the MIRU-VNTR assay protocol from Castro-Rodríguez et al. (7) (performed at the research laboratories of Universidad de Las Américas (Quito, Ecuador)) and conducted it in a final volume of 15 μL. We prepared a master mix for each of the 24-locus containing 7.5 μL GoTaq Green Master Mix (1X), 0.75 μL each of the forward and reverse primers, 1 μL genomic DNA, and 5 μL nuclease-free water to a final volume of 15 μL. Thermocycling conditions were as follows: initial denaturation at 94 °C for 5 min; 35 cycles of denaturation at 94 °C for 1 min, annealing at 54 °C for 1 min 30 s, and extension at 72 °C for 1 min 30 s; followed by a final extension at 72 °C for 10 min. We resolved PCR amplicons on 2% agarose gels run at 85 V for 3 h, using the same 100 bp molecular weight ladder. We evaluated bands to determine allele sizes at each locus, yielding a 24-digit MIRU-VNTR profile per sample.

Genotyping followed the standardized 24-locus MIRU-VNTR scheme of Supply et al. (12), using an in-house protocol; no commercial kit was used. The 24 loci were amplified individually using the primer pairs listed in Supplementary Table 1.

### 2.4 Spoligotyping assays

We performed spoligotyping according to Kamerbeek et al. (13) at the Laboratory of Animal Health, University of Extremadura. The method is based on the presence or absence of 43 spacer sequences interspersed between conserved loci in the Direct Repeat (DR) region. The DR region was amplified by PCR using the primer DRa (5′-GGTTTTGGGTCTGACGAC-3′, 5′-biotinylated) and primer DRb (5′-CCGAGAGGGGACGGAAAC-3′). We hybridized the biotinylated PCR products to a commercial membrane containing 43 immobilized oligonucleotide probes, each corresponding to a unique spacer sequence within the DR locus. After hybridization, we washed the membrane and incubated it with a streptavidin–peroxidase conjugate (Roche, USA). We detected the hybridized DNA using chemiluminescent Amersham ECL reagents (GE Healthcare, UK) and visualized it by exposure to Hyperfilm ECL (Merck, USA) in an X-ray cassette; we then developed and fixed the film for interpretation. We recorded the resulting presence/absence signal for each spacer in binary and octal formats, and assigned lineages by comparison with the Fourth International Spoligotyping Database (SpolDB4).

### 2.5 Whole-genome sequencing (WGS) and bioinformatic analysis

We extracted genomic DNA from RD^Rio^-positive *M. tuberculosis* isolates from cultures using the PureLink Genomic DNA Mini Kit (Invitrogen, Thermo Fisher Scientific, USA), following the manufacturer’s instructions. We prepared sequencing libraries using the Illumina DNA Prep kit (Illumina, San Diego, CA, USA) and performed whole-genome sequencing on an Illumina MiSeq platform at Biosequence S. A. (Quito, Ecuador), generating 2 × 150 bp paired-end reads. Raw FASTQ files showed high sequencing quality, with over 85% of bases having a Phred quality score ≥30 (Q30), and were therefore used directly as input for downstream analysis without additional manual quality trimming.

Raw reads were processed using TBProfiler (version 6.6.5) (14), which performs internal read trimming and alignment to the *M. tuberculosis* H37Rv reference genome (NC_000962.3) prior to variant calling. Median genome coverage, as calculated by TBProfiler, ranged from 36X to 91X across isolates. We determined phylogenetic lineage and sublineage assignment using TBProfiler’s barcoding scheme, based on a validated set of lineage-defining SNPs (14), and inferred genotypic drug resistance profiles using the default TBProfiler mutation database (tbdb; commit 0a909094), which incorporates resistance-associated variants cataloged by the World Health Organization.

### 2.6 Minimum spanning tree analysis

We performed minimum spanning tree (MST) analyses using MIRU-VNTR 24-locus and spoligotyping profiles from Ecuadorian RD^Rio^ isolates identified by Duplex PCR in this study and from previously isolated isolates reported by Castro-Rodriguez et al. (7). We also performed an additional MST analysis using 24-locus MIRU-VNTR data. We incorporated international RD^Rio^ reference isolates from different geographic regions to provide a global phylogeographic context. We organized allelic profiles into a tab-delimited dataset containing isolate identifiers and corresponding locus values. We incorporated epidemiological metadata, including country of origin, province, year of isolation, and antibiotic resistance status, as auxiliary data for visualization. Supplementary Table S2 provides the complete set of 24-locus MIRU-VNTR and spoligotyping profiles used in these analyses, along with the associated epidemiological metadata.

We generated MSTs using the goeBURST algorithm (15). We inferred genetic relationships among isolates from allelic differences at the MIRU-24 and spoligotyping loci. Nodes in the network represented unique profiles, while edges corresponded to the number of allelic variations between connected isolates. We produced distinct visualizations by coloring nodes based on epidemiological variables, enabling exploratory assessment of clustering patterns and genetic relatedness rather than definitive phylogenetic reconstructions.

Because MIRU-VNTR and spoligotyping are based on allelic profile comparisons rather than whole-genome sequence data, we interpreted the resulting MSTs as representations of genetic relatedness and epidemiological clustering rather than definitive phylogenetic reconstructions.

### 2.7 Statistical analysis

We assessed differences in RD^Rio^ prevalence between the two study years (2018 and 2023) using the chi-square test with Yates’ continuity correction. We calculated exact 95% confidence intervals for prevalence estimates using the Wilson score method. We considered a two-sided *p*-value < 0.05 statistically significant. All analyses were performed in Python v3.12.3 using the SciPy (v1.17.1) and statsmodels (v0.15.0) libraries.

## 3 Results

### 3.1 Prevalence of the RD^Rio^ genotype

We screened 699 *M. tuberculosis* clinical isolates collected in Ecuador between 2018 (384 strains) and 2023 (315 strains) for the RD^Rio^ genotype by duplex PCR. Of these, 40 isolates yielded an amplification pattern consistent with the RD^Rio^ deletion, corresponding to an overall prevalence of 5.72% (95% CI, 4.23–7.70%) in the analyzed MTB population. RD^Rio^ isolates were distributed across both study years, with 20 identified among isolates collected in 2018 (5.21%; 95% CI, 3.40–7.91%) and 20 among isolates collected in 2023 (6.35%; 95% CI, 4.15–9.60%). This difference between years was not statistically significant (*χ*^2^ test with Yates’ correction, *p* = 0.63). Notably, three isolates showed simultaneous amplification of both the wild-type (530-bp) and RDRio-specific (1175-bp) PCR products, consistent with co-infection by RD^Rio^ and non-RDRio (wild-type) *M. tuberculosis* strains within the same sample.

Figure 1 shows the geographic distribution of the 40 RD^Rio^-positive isolates identified in the present study across Ecuadorian provinces, with the highest numbers in Guayas (*n* = 11) and Los Ríos (*n* = 11).

**Figure 1:**
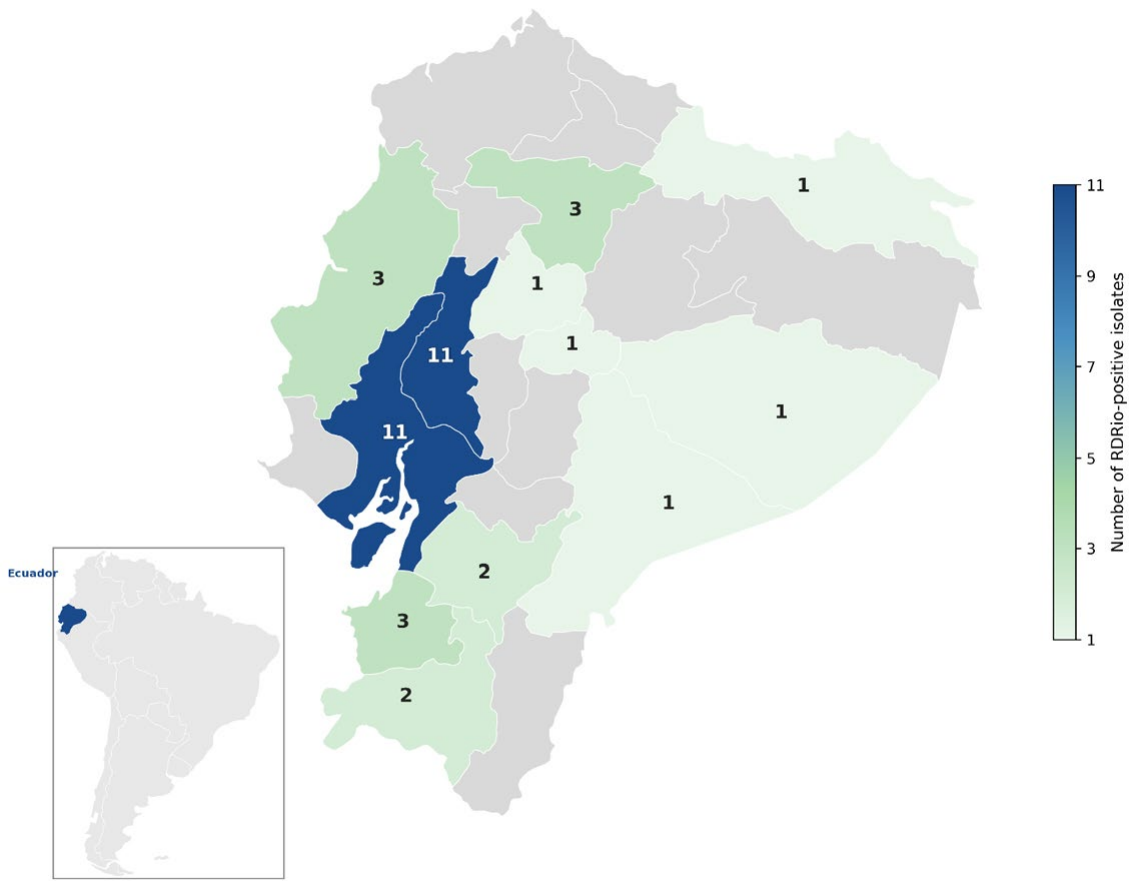
Geographic distribution across Ecuadorian provinces of the 40 RD^Rio^-positive *M. tuberculosis* isolates identified in the present study (2018 and 2023) by duplex PCR. Color intensity indicates the number of RD^Rio^-positive isolates per province, as shown by the scale; the number within each shaded province corresponds to that count. Provinces shown in grey had no RD^Rio^-positive isolates. From west to east, the provinces with RD^Rio^-positive isolates were Guayas (*n* = 11), Los Ríos (*n* = 11), Manabí (*n* = 3), El Oro (*n* = 3), Pichincha (*n* = 3), Azuay (*n* = 2), Loja (*n* = 2), Cotopaxi (*n* = 1), Tungurahua (*n* = 1), Morona Santiago (*n* = 1), Pastaza (*n* = 1), and Sucumbíos (*n* = 1). The inset shows Ecuador’s location within South America.

### 3.2 Genotypic diversity of RD^Rio^ isolates by MIRU-VNTR and spoligotyping

We genotyped the 40 RD^Rio^-positive isolates using 24-locus MIRU-VNTR typing and spoligotyping. Interpretable 24-locus MIRU-VNTR profiles were obtained for all 40 isolates; using the automated lineage-assignment function of the MIRU-VNTRplus platform, 23 isolates were identified as LAM, 3 as Haarlem, 2 as S-type, and 1 as Uganda I, while 11 isolates returned a best-match distance above the similarity threshold of the MIRU-VNTRplus automated identification function and were therefore left unassigned by the algorithm, rather than representing orphan patterns absent from the reference database. We performed spoligotyping on 32 of the 40 isolates; we could not test the remaining 8 because of insufficient DNA. Among the 32 isolates tested, spoligotyping-based lineage assignment identified 28 as LAM, 1 as S-type, 1 as TUR, 1 as Uganda II/Ghana, and 1 as West African 1. When MIRU-VNTR and spoligotyping data were consolidated within the MIRU-VNTRplus platform to generate a single combined lineage assignment per isolate, 36 of the 40 RD^Rio^-positive isolates (90.0%) were successfully identified: 32 as LAM, 2 as Haarlem, 1 as S-type, and 1 as Uganda I. The other 4 isolates (10.0%) remained unassigned by the MIRU-VNTRplus algorithm under both methods.

### 3.3 WGS-based sublineage assignment and genotypic drug resistance profile

We performed whole-genome sequencing on 28 of the 40 RD^Rio^-positive isolates. TBProfiler-based sublineage assignment identified 16 isolates as sublineage 4.3.4.1, six as 4.3.4.2, two as 4.3.4, and one as 4.3.3; these sublineages belonged to Lineage 4 (Euro-American), consistent with the LAM designation; 1 was 4.4.1.1 (S-type); and 2 were 4.1.1 (X-type). Two isolates showed sequencing evidence of mixed infection, with reads supporting the simultaneous presence of sublineages 4.3.4.1 (LAM) and 4.4.1.1 (S-type) in one isolate, and 4.1.1 (X-type) and 4.3.4.1 (LAM) in the other.

We predicted genotypic drug-resistance profiles from WGS data using TBProfiler for the 28 sequenced isolates. Based on these predictions, we classified 12 isolates (42.9%) as pan-susceptible, 4 (14.3%) as genotypically isoniazid-resistant, rifampicin-susceptible TB (Hr-TB), 9 (32.1%) as predicted multidrug-resistant TB (MDR-TB), and 2 (7.1%) as predicted pre-extensively drug-resistant TB (pre-XDR-TB). One additional isolate (3.6%) carried mutations predicting fluoroquinolone resistance (moxifloxacin and levofloxacin) in the absence of rifampicin or isoniazid resistance and, as this pattern does not meet the WHO criteria for MDR/RR-TB or pre-XDR-TB, is reported here as predicted fluoroquinolone-resistant, non-MDR/RR-TB. As phenotypic drug-susceptibility testing was not available for these isolates, all resistance classifications reported here are genotypic predictions and were not phenotypically confirmed. Supplementary Table S2 lists the specific resistance-associated mutations underlying each classification.

Table 1 presents individual-level genotyping results for all 40 RD^Rio^-positive isolates, along with WGS-derived sublineage and genotypic drug-resistance profiles for the sequenced subset.

**Table 1:** Genotypic and whole-genome sequencing (WGS)–based characterization of RD^Rio^-positive *Mycobacterium tuberculosis* isolates identified in Ecuador, 2018 and 2023 (*n* = 40).

No.	Sample ID	Isolate ID	SRA accession(s)	Year	MIRU-VNTR	Spoligotyping	MIRU-VNTR + spoligotyping	Strain type (WGS)	Family (WGS)	Genotypic drug resistance (WGS-predicted)
1	BBH 801	ECU_RDRio_25	SRR40532564, SRR40532563	2018	S-type	–	S-type	4.4.1.1	S-type	MDR-TB
2	VAL 256	ECU_RDRio_19	–	2018	LAM	–	LAM	–	–	–
3	PORTO 202	ECU_RDRio_20	–	2018	S	–	LAM	–	–	–
4	MACAS 799	ECU_RDRio_24	SRR40532548	2018	LAM	LAM	LAM	4.3.4.2	LAM	Hr-TB
5	BBH 1333 (4)	ECU_RDRio_33	SRR40532618	2018	LAM	LAM	LAM	4.3.4.2	LAM	Pre-XDR-TB
6	VAL 1169	ECU_RDRio_35	SRR40532616	2018	LAM	LAM	LAM	4.3.4.2	LAM	Hr-TB
7	BBH 1333 (5)	ECU_RDRio_37	SRR40532614	2018	LAM	LAM	LAM	4.3.4.2	LAM	Pre-XDR-TB
8	647	ECU_RDRio_49	SRR40532546	2023	LAM	TUR	LAM	4.3.4.2	LAM	Susceptible
9	1,010	ECU_RDRio_55	SRR40532611	2023	?	LAM	LAM	4.3.4.2	LAM	Hr-TB
10	V 908	ECU_RDRio_28	SRR40532557, SRR40532556	2018	?	–	–	4.3.4.1; 4.4.1.1	LAM; S-type	MDR-TB
11	MACH 11	ECU_RDRio_21	SRR40532560, SRR40532559	2018	UGANDA I	LAM	UGANDA I	4.3.4.1	LAM	Hr-TB
12	VAL 920	ECU_RDRio_29	–	2018	LAM	–	LAM	–	–	–
13	MACH 847	ECU_RDRio_26	SRR40532621	2018	LAM	LAM	LAM	4.3.4.1	LAM	MDR-TB
14	BBH 1061	ECU_RDRio_31	–	2018	Haarlem	–	Haarlem	–	–	–
15	BBH 1480	ECU_RDRio_32	–	2018	LAM	–	LAM	–	–	–
16	CCA 874	ECU_RDRio_27	SRR40532620	2018	LAM	LAM	LAM	4.3.4.1	LAM	MDR-TB
17	VAL 1331 (4)	ECU_RDRio_34	SRR40532617	2018	LAM	LAM	LAM	4.3.4.1	LAM	MDR-TB
18	VAL 1331 (5)	ECU_RDRio_36	SRR40532615	2018	?	LAM	LAM	4.3.4.1	LAM	MDR-TB
19	1,345	ECU_RDRio_38	SRR40532613	2023	LAM	LAM	LAM	4.3.4.1	LAM	Susceptible
20	1,252	ECU_RDRio_42	SRR40532612	2023	?	LAM	LAM	4.3.4.1	LAM	Susceptible
21	1,385	ECU_RDRio_45	SRR40532552	2023	?	LAM	LAM	4.3.4.1	LAM	Susceptible
22	609	ECU_RDRio_46	SRR40532551	2023	?	LAM	LAM	4.3.4.1	LAM	Susceptible
23	1,003	ECU_RDRio_40	–	2023	LAM	–	LAM	–	–	–
24	612	ECU_RDRio_47	SRR40532550	2023	?	LAM	LAM	4.3.4.1	LAM	Susceptible
25	678	ECU_RDRio_51	SRR40532549	2023	?	LAM	LAM	4.3.4.1	LAM	Susceptible
26	1,255	ECU_RDRio_43	–	2023	LAM	–	LAM	–	–	–
27	1,064	ECU_RDRio_53	SRR40532638, SRR40532637	2023	LAM	LAM	LAM	4.3.4.1	LAM	Susceptible
28	501	ECU_RDRio_56	SRR40532636, SRR40532635	2023	LAM	LAM	LAM	4.3.4.1	LAM	FQ-resistant, non-MDR/RR-TB
29	502	ECU_RDRio_58	SRR40532632, SRR40532631	2023	Haarlem	LAM	LAM	4.3.4.1	LAM	Susceptible
30	UIO 572	ECU_RDRio_18	SRR40532623	2018	LAM	LAM	LAM	4.3.4	LAM	MDR-TB
31	QUI 17	ECU_RDRio_22	SRR40532622	2018	LAM	LAM	LAM	4.3.4	LAM	MDR-TB
32	1,243	ECU_RDRio_41	SRR40532555, SRR40532554	2023	?	LAM	–	4.3.3	LAM	Susceptible
33	630	ECU_RDRio_48	SRR40532577	2023	?	Uganda II/Ghana	–	4.1.1; 4.3.4.1	X-type; LAM	Susceptible
34	VAL 1089	ECU_RDRio_30	SRR40532562, SRR40532561	2018	Haarlem	–	Haarlem	4.1.1	X-type	MDR-TB
35	681	ECU_RDRio_52	SRR40532566, SRR40532565	2023	?	S-type	–	4.1.1	X-type	Susceptible
36	VAL 371	ECU_RDRio_23	–	2018	LAM	LAM	LAM	–	–	–
37	1,088	ECU_RDRio_39	–	2023	LAM	West African 1	LAM	–	–	–
38	1,260	ECU_RDRio_44	–	2023	LAM	LAM	LAM	–	–	–
39	651	ECU_RDRio_50	–	2023	LAM	LAM	LAM	–	–	–
40	1,009	ECU_RDRio_54	–	2023	LAM	LAM	LAM	–	–	–

We determined sample-level lineage assignment independently by 24-locus MIRU-VNTR typing and spoligotyping, and by their consolidated assignment (MIRU-VNTRplus). We determined strain type, family, and genotypic drug-resistance profiles by WGS and inferred them using TBProfiler for the subset of sequenced isolates (*n* = 28/40). Isolate ID corresponds to the identifier used across figures and Supplementary Table S2; SRA accessions correspond to NCBI BioProject PRJNA1524051 (two accessions indicate re-sequenced samples). “–,” test not performed (MIRU-VNTR/spoligotyping: insufficient DNA; WGS columns: isolate not sequenced); “?,” test performed but the profile remained unassigned by MIRU-VNTRplus. All drug-resistance classifications are genotypic predictions (WGS/TBProfiler) and were not phenotypically confirmed. Hr-TB, isoniazid-resistant, rifampicin-susceptible TB; MDR-TB, multidrug-resistant TB; Pre-XDR-TB, pre-extensively drug-resistant TB; FQ-resistant non-MDR/RR-TB, isolated fluoroquinolone resistance not meeting MDR/RR-TB criteria. The fluoroquinolone-resistant isolate (Sample ID 501) showed gyrA heteroresistance (A90V and D94N); we characterized it using TBProfiler v6.7.0 (who_v2 + database), whereas all other isolates used TBProfiler v6.6.5 (tbdb commit 0a909094).

### 3.4 MST analysis of RD^Rio^ strains isolated in Ecuador

To assess the genetic relatedness of RD^Rio^ isolates identified in Ecuador, we performed an MST analysis using 24-loci MIRU-VNTR profiles from RD^Rio^-positive isolates identified in the present study (2018 and 2023), combined with those reported in the 2012–2016 baseline study (7). Of 57 isolates initially included, we retained 49 for analysis after excluding isolates with more than four loci with missing (NA) data.

When isolates were labeled according to study of origin (Figure 2A), two main clonal complexes were identified within the MST: one composed predominantly of isolates from the present study (2018 and 2023), and the other composed predominantly of isolates from the 2012–2016 baseline collection, with a smaller number of isolates from each period distributed peripherally or in proximity to the complex predominantly associated with the other study. When the same MST was labeled by province of origin (Figure 2B), isolates originated from 14 Ecuadorian provinces. Guayas (*n* = 21) and Los Ríos (*n* = 9) accounted for most isolates, while the remaining 19 isolates were distributed across 12 additional provinces (Pichincha, Manabí, El Oro, Santo Domingo de los Tsáchilas, Azuay, Loja, Esmeraldas, Santa Elena, Morona Santiago, Sucumbíos, Cotopaxi, and Pastaza). These province counts refer to the combined dataset of 49 isolates (present study and reference 7) retained after the NA-exclusion criterion and therefore differ from the province distribution of the 40 isolates identified in the present study alone (Figure 1). Isolates from Guayas and Los Ríos provinces were each concentrated within distinct regions of the MST, whereas isolates from the remaining provinces did not display a clearly defined geographical clustering pattern.

**Figure 2:**
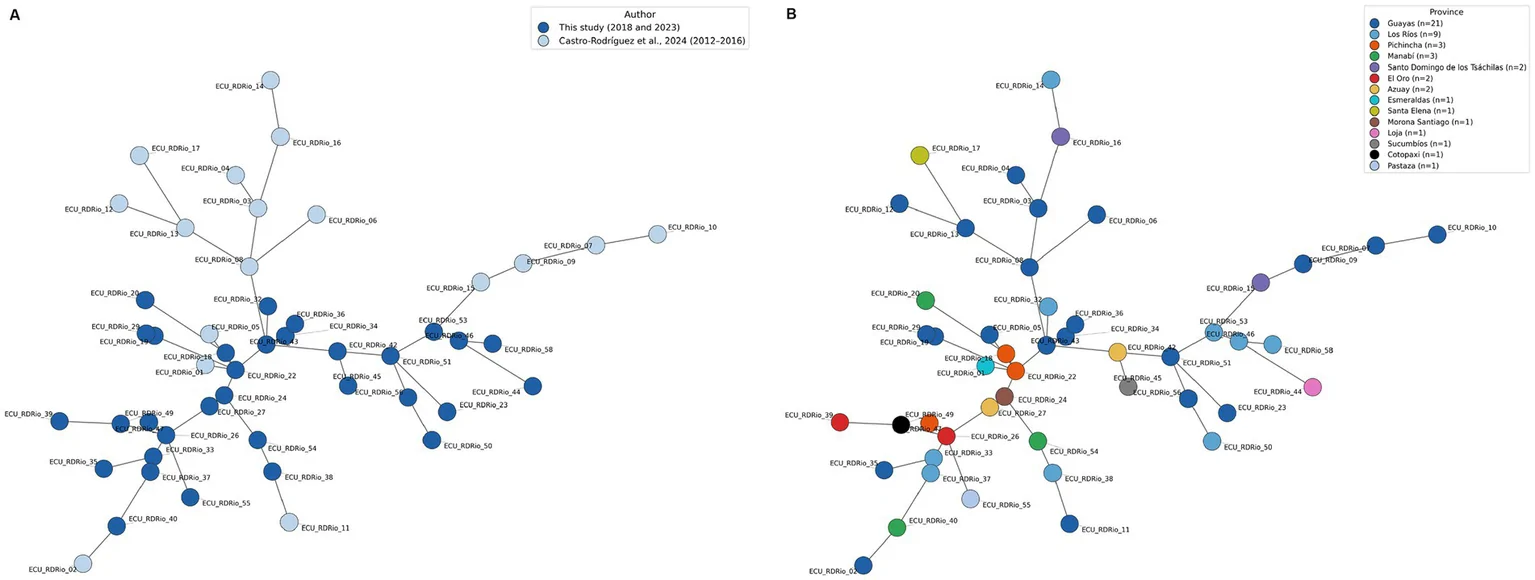
MST of MTB RD^Rio^ isolates from Ecuador, based on 24-loci MIRU-VNTR profiles, combining isolates from the present study (2018–2023) with those from the 2012–2016 baseline study (7). Of 57 isolates initially analyzed, we retained 49 after excluding isolates with ≥4 loci with missing (NA) data. Each node represents a unique MIRU-VNTR profile, and edges indicate the number of allelic differences between connected profiles. **(A)** Nodes are colored according to years (dark blue: 2018–2023; light blue: 2012–2016). **(B)** Nodes are colored by province of origin. We generated the MST using the goeBURST algorithm.

To place the RD^Rio^ isolates identified in Ecuador within a broader international context, a second MST analysis was performed using 24-loci MIRU-VNTR profiles, combining the 49 Ecuadorian RDRio isolates described above with 61 international reference isolates retrieved from previously published studies, representing Brazil (*n* = 19), Portugal (*n* = 17), Russia (*n* = 7), Colombia (*n* = 1), China (*n* = 1), and Venezuela (*n* = 16) (8, 16, 17) (Figure 3). When nodes were labeled by country of origin, most Ecuadorian isolates clustered within a single, extensive MST branch that did not include isolates from any other country. Fewer Ecuadorian isolates were distributed among several smaller peripheral clusters, where they were intermixed with isolates from Brazil, Portugal, Russia, Colombia, China, and Venezuela (Figure 3).

**Figure 3:**
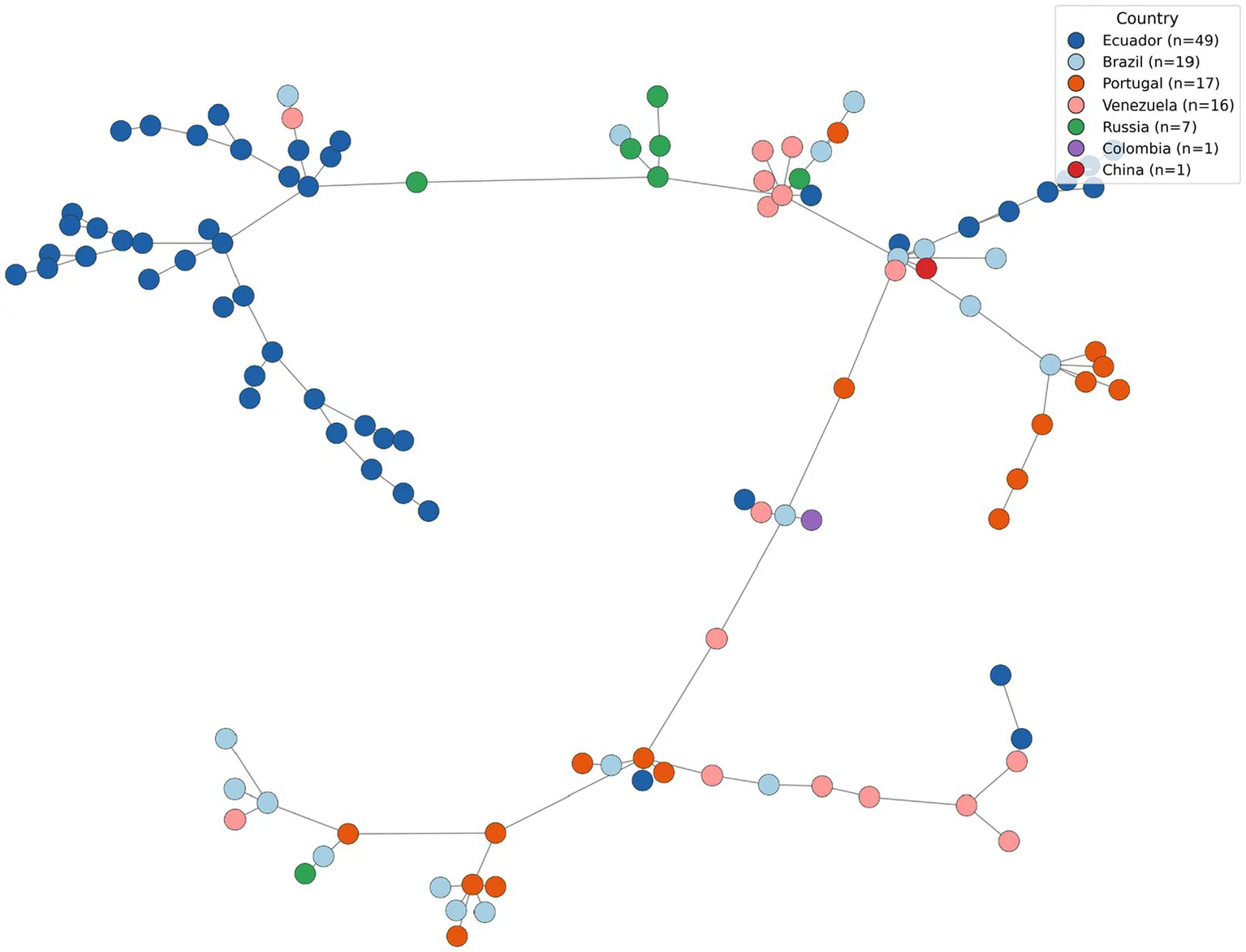
MST of *M. tuberculosis* RDRio isolates based on 24-locus MIRU-VNTR profiles, combining 49 Ecuadorian RDRio isolates (this study and reference 7) with 61 international reference isolates from Brazil, Portugal, Russia, Colombia, China, and Venezuela, retrieved from previously published studies (8, 16, 18). Each node represents a unique MIRU-VNTR profile, and edges indicate the number of allelic differences between connected profiles. Nodes are colored by country of origin. We generated the MST using the goeBURST Full MST algorithm implemented in Python.

## 4 Discussion

In this study, we provide an updated molecular epidemiological assessment of the RD^Rio^ genotype of *M. tuberculosis* in Ecuador for 2018 and 2023, building on the 2012–2016 baseline established previously in our laboratory (7). We identified an overall RD^Rio^ prevalence of 5.72% across 2018–2023 (5.21% in 2018 and 6.35% in 2023, a difference that was not statistically significant; *χ*^2^ test, *p* = 0.63), representing a nearly three-fold increase compared to the 2.09% reported for the 2012–2016 period (7). While this rise is notable, the prevalence of RD^Rio^ in Ecuador remains substantially lower than that reported in neighboring high-endemicity settings such as Venezuela, where up to 69.8% of LAM isolates belong to the RD^Rio^ genotype (8), and is comparable to or below values reported in other South American countries (4). Several non-mutually exclusive factors may contribute to the observed increase, including genuine epidemiological change and differences in the geographic composition of the sampled collections between the two study periods. Because both studies drew isolates from the same national reference laboratory in Guayaquil, we acknowledge a shared geographic sampling bias toward Coastal provinces when interpreting this temporal comparison (7).

The consolidated MIRU-VNTR and spoligotyping analysis assigned most RD^Rio^-positive isolates to the LAM family (32/40; 80.0%), consistent with RD^Rio^’s well-established membership in LAM. However, a subset of isolates could not be assigned to a defined lineage by either classical method, and a small number were assigned to non-LAM families such as Haarlem, S-type, Uganda I, and others. This apparent heterogeneity is consistent with prior reports showing that spoligotyping, in particular, has limited discriminatory power within the Euro-American lineage because of convergent evolution (homoplasy) in the direct-repeat region and the high number of IS6110 copies characteristic of these strains (18). Notably, the RD^Rio^ genotype is not strictly confined to canonical LAM spoligotype patterns: studies incorporating additional markers have shown that spoligotyping alone may misclassify RD^Rio^ strains as non-LAM, underscoring the value of complementary typing approaches (16, 18).

Our WGS-based sublineage assignments help resolve some of this ambiguity. WGS of 28 RD^Rio^ genotype strains confirmed that the dominant sublineages were 4.3.4.1 and 4.3.4.2, both nested within Lineage 4 (Euro-American), consistent with the LAM designation obtained by classical typing and providing high-resolution validation of the genotyping workflow. Importantly, WGS also revealed sublineage assignments that classical methods could not fully resolve, including isolates classified as 4.4.1.1 (S-type) and 4.1.1 (X-type), as well as two isolates with evidence of mixed infection. Because this study did not include phenotypic drug-susceptibility testing for the RDRio-positive isolates—unlike the baseline study, which had access to phenotypic resistance data (7)—TBProfiler-based genotypic resistance prediction served as a necessary compensatory source of resistance information. This is particularly relevant because genotype-based prediction shows high concordance with phenotypic results for the first-line drugs most relevant to MDR classification (isoniazid and rifampicin), while concordance is lower for other agents and genomic prediction remains best suited to confirming resistance rather than susceptibility (19, 20). We deliberately limited this genomic component; future studies will include deeper phylogenomic characterization, including cgSNP-based transmission analysis and integration of additional sequenced isolates from earlier periods.

Genotypic resistance profiling classified 32.1% (9/28) of sequenced isolates as MDR-TB and 7.1% (2/28) as pre-XDR-TB, with an additional isolated case of fluoroquinolone resistance. The proportion of MDR among sequenced RD^Rio^ isolates is broadly consistent with the strong association between the RD^Rio^ genotype and multidrug resistance repeatedly documented in the literature, particularly in Brazil and in the previous Ecuadorian study (4, 6, 7). This association may reflect enhanced biological fitness or transmissibility of RDRio strains—potentially linked to deletion of the *PPE55*/*PPE56* genes and reduced host immune recognition—which may favor the propagation of resistant clones (4, 5). While our sample size precludes firm epidemiological inference, the persistence of a high resistance burden among RD^Rio^ isolates reinforces the relevance of continued molecular surveillance of this genotype in Ecuador.

MST analysis based on MIRU-VNTR profiles revealed that RD^Rio^ isolates from 2018 and 2023, and from 2012 to 2016, segregated predominantly into distinct clonal complexes. When the analysis was restricted to a single province (Guayas), for which isolates were available from both periods, the dominant complexes of each period remained largely distinct, suggesting that this temporal segregation is not fully explained by differences in the geographic composition of the two collections and may reflect a shift in the predominant circulating RD^Rio^ clonal complex over time. Nevertheless, partial overlap between complexes indicates that this apparent turnover was not absolute, and co-circulation of related complexes cannot be excluded. Consistent with the baseline study, we observed a regional concentration of RD^Rio^ isolates in Coastal provinces (notably Guayas and Los Ríos) alongside considerable genetic diversity and a lack of extensive clustering, a pattern indicative of high diversity with limited active transmission chains (7).

To place the Ecuadorian isolates within a broader context, we incorporated international RDRio reference profiles from Brazil, Portugal, Russia, Colombia, China, and Venezuela (8, 16, 18). The dominant Ecuadorian clonal complex remained largely isolated from all international reference clusters—including Venezuelan isolates specifically selected to represent distinct circulating clades—whereas only a smaller subset of Ecuadorian isolates intermixed with international strains. This pattern is noteworthy considering the hypothesis that the increasing RDRio burden in Ecuador might be driven by recent Venezuelan diaspora, where RDRio is highly endemic (8); since 2015, and as of July 2023, at least 502,200 Venezuelan refugees and migrants have been recorded in Ecuador, concentrated precisely in the western provinces where our isolates predominate (9). The genetic isolation of the dominant Ecuadorian complex from the Venezuelan reference strains suggests that this complex may represent a more established or under-sampled local lineage rather than a signature of recent importation, while the smaller intermixed subset is more compatible with transnational introduction. This interpretation aligns with broader phylogeographic evidence that successive waves of human migration over long timescales have shaped the global dissemination of the LAM lineage, including the RD174/RDRio sublineage (18).

This study has several limitations. First, phenotypic drug-susceptibility testing was not available, so we inferred resistance profiles solely from WGS; although genotypic prediction is highly concordant with phenotypic results for isoniazid and rifampicin, it is not equivalent across all drugs. Second, we could not perform spoligotyping and WGS on all isolates (10/40 and 12/40, respectively), mainly because of insufficient DNA, which may introduce selection effects. Third, we used WGS data only to infer genotypic resistance and confirm sublineage assignment, not for genome-wide phylogenetic reconstruction; we therefore assessed genetic relationships using MIRU-VNTR and spoligotyping, which offer lower resolution than core-genome SNP (cgSNP) analysis and are subject to homoplasy. The MST clustering patterns should thus be read as representations of genetic relatedness rather than definitive evidence of transmission or phylogeny, and the international and baseline comparisons are additionally subject to sampling and geographic biases inherent to retrospective, multi-source datasets. Finally, the analyzed isolates correspond to culture-positive strains referred to and preserved at INSPI—predominantly from Coastal provinces—rather than all notified TB cases in Ecuador (6,094 in 2018 and 8,278 in 2023, according to national MSP reports (21, 22)); isolates from the intervening years (2019–2022) were not processed owing to budget and infrastructure constraints, leaving the dynamics of those years uncharacterized. A comprehensive cgSNP-based phylogenomic characterization of RD^Rio^ in Ecuador, integrating additional sequenced and international reference genomes, is currently in preparation as a dedicated study.

In conclusion, the prevalence of the MTB RD^Rio^ genotype in Ecuador remains relatively low, especially compared to neighboring countries like Venezuela. Our results did not support a strong importation of RD^Rio^ strains associated with the recent massive Venezuelan migration to Ecuador. Nevertheless, despite the low prevalence, the association of the RD^Rio^ genotype with drug-resistant TB suggests that periodically monitoring these strains would reinforce TB surveillance and control strategies in Ecuador.

## Data Availability

The datasets presented in this study can be found in online repositories. The names of the repository/repositories and accession number(s) can be found in the article/Supplementary material.
