# Supplementary material for: Molecular epidemiology of the RDRio genotype of Mycobacterium tuberculosis in Ecuador: a tracking update for the years 2018 and 2023
Source: Front Public Health. 2026 Oct 27;14:1969751. doi: 10.3389/fpubh.2026.1969751 (PMC13748437; doi:10.3389/fpubh.2026.1969751)
Supplement: Supplementary file 1 [file Table_1.DOCX]

**Supplementary Table 1. Primer sequences used for the 24-locus MIRU-VNTR genotyping.**

| **Locus alias** | **Locus (alias no.)** | **Forward primer (5′→3′)** | **Reverse primer (5′→3′)** |
| --- | --- | --- | --- |
| MIRU02 | 154 | TGGACTTGCAGCAATGGACCAACT | TACTCGGACGCCGGCTCAAAAT |
| Mtub04 | 424 | CTTGGCCGGCATCAAGCGCATTATT | GGCAGCAGAGCCCGGGATTCTTC |
| ETRC | 577 | CGAGAGTGGCAGTGGCGGTTATCT | AATGACTTGAACGCGCAAATTGTGA |
| MIRU04 | 580 | GCGCGAGAGCCCGAACTGC | GCGCAGCAGAAACGCCAGC |
| MIRU40 | 802 | GGGTTGCTGGATGACAACGTGT | GGGTGATCTCGGCGAAATCAGATA |
| MIRU10 | 960 | GTTCTTGACCAACTGCAGTCGTCC | GCCACCTTGGTGATCAGCTACCT |
| MIRU16 | 1644 | TCGGTGATCGGGTCCAGTCCAAGTA | CCCGTCGTGCAGCCCTGGTAC |
| Mtub21 | 1955 | CGAAGTGAATGGTGGCAT | CAACATCGCCTGGTTCTGTA |
| MIRU20 | 2059 | TCGGAGAGATGCCCTTCGAGTTAG | GGAGACCGCGACCAGGTACTTGTA |
| QUB11b | 2163b | CGTAAGGGGGATGCGGGAAATAGG | CGAAGTGAATGGTGGCAT |
| ETRA | 2165 | AAATCGGTCCCATCACCTTCTTAT | CGAAGCCTGGGGTGCCCGCGATTT |
| Mtub29 | 2347 | GCCAGCCGCCGTGCATAAACCT | AGCCACCCGGTGTGCCTTGTATGAC |
| Mtub30 | 2401 | CTTGAAGCCCCGGTCTCATCTGT | ACTTGAACCCCCACGCCCATTAGTA |
| ETRB | 2461 | ATGGCCACCCGATACCGCTTCAGT | CGACGGGCCATCTTGGATCAGCTAC |
| MIRU23 | 2531 | CTGTCGATGGCCGCAACAAAACG | AGCTCAACGGGTTCGCCCTTTTGTC |
| MIRU24 | 2687 | CGACCAAGATGTGCAGGAATACAT | GGGCGAGTTGAGCTCACAGAA |
| MIRU26 | 2996 | TAGGTCTACCGTCGAAATCTGTGAC | CATAGGCGACCAGGCGAATAG |
| MIRU27 | 3007 | TCGAAAGCCTCTGCGTGCCAGTAA | GCGATGTGAGCGTGCCACTCAA |
| Mtub34 | 3171 | GGTGCGCACCTGCTCCAGATAA | GGCTCTCATTGCTGGAGGGTTGTAC |
| MIRU31 | 3192 | ACTGATTGGCTTCATACGGCTTTA | GTGCCGACGTGGTCTTGAT |
| Mtub39 | 3690 | CGGTGGAGGCGATGAACGTCTTC | TAGAGCGGCACGGGGGAAAGCTTAG |
| QUB26 | 4052 | AACGCTCAGCTGTCGGAT | CGGCCGTGCCGGCCAGGTCCTTCCCGAT |
| QUB4156 | 4156 | TGACCACGGATTGCTCTAGT | GCCGGCGTCCATGTT |
| MIRU39 | 4348 | CGCATCGACAAACTGGAGCCAAAC | CGGAAACGTCTACGCCCCACACAT |

The 24 loci were amplified in individual reactions following the standardized 24-locus MIRU-VNTR scheme of Supply et al. (2006), using an in-house protocol; no commercial genotyping kit was used. Loci are listed in the order used for the 24-digit MIRU-VNTR profile, with their conventional alias and alias number. Amplicon sizes were resolved by agarose gel electrophoresis against a 100-bp molecular-weight marker (Opti-DNA Marker), and fragment sizes were converted to repeat numbers at each locus.
