# Supplementary material for: Molecular epidemiology of the RDRio genotype of Mycobacterium tuberculosis in Ecuador: a tracking update for the years 2018 and 2023
Source: Front Public Health. 2026 Oct 27;14:1969751. doi: 10.3389/fpubh.2026.1969751 (PMC13748437; doi:10.3389/fpubh.2026.1969751)
Supplement: Supplementary file 2 [file Table_3.DOCX]

**Supplementary Table 3. Genotypic drug-resistance predictions and resistance-associated mutations for the WGS-sequenced RD^Rio^-positive isolates (n = 28).**

| **n°** | **Sample ID** | **Isolate ID** | **SRA accession(s)** | **Sublineage (TBProfiler)** | **Predicted resistance** | **Predicted resistant drugs** | **Resistance-associated mutation(s) [gene]** |
| --- | --- | --- | --- | --- | --- | --- | --- |
| 1 | BBH 801 | ECU_RDRio_25 | SRR40532564, SRR40532563 | 4.4.1.1 | MDR-TB | Rifampicin, Rifapentine, Isoniazid, Pirazinamide, Streptomycin | p.Ser450Leu (rpoB - Rifampicin, Rifapentine), p.Ser315Thr (katG - Isoniazid), p.Gly24Asp (pncA - Pyrazinamide), p.Lys43Arg (rpsL - Streptomycin) |
| 4 | MACAS 799 | ECU_RDRio_24 | SRR40532548 | 4.3.4.2 | Hr-TB | Isoniazid | p.Ser315Thr (katG - Isoniazid) |
| 5 | BBH 1333 (4) | ECU_RDRio_33 | SRR40532618 | 4.3.4.2 | Pre-XDR-TB | Rifampicin, Rifapentine, Isoniazid, Ethambutol, Moxifloxacin, Levofloxacin, Streptomycin | p.His445Leu (rpoB - Rifampicin, Rifapentine), p.Ser315Thr (katG - Isoniazid), p.Met306Ile (embB - Ethambutol), p.Asp94Gly (gyrA - Moxifloxacin, Levofloxacin), p.Lys88Arg (rpsL - Streptomycin) |
| 6 | VAL 1169 | ECU_RDRio_35 | SRR40532616 | 4.3.4.2 | Hr-TB | Isoniazid | p.Ser315Thr (katG - Isoniazid) |
| 7 | BBH 1333 (5) | ECU_RDRio_37 | SRR40532614 | 4.3.4.2 | Pre-XDR-TB | Rifampicin, Rifapentine, Isoniazid, Ethambutol, Moxifloxacin, Levofloxacin, Streptomycin | p.His445Leu (rpoB - Rifampicin, Rifapentine), p.Ser315Thr (katG - Isoniazid), p.Met306Ile (embB - Ethambutol), p.Asp94Gly (gyrA - Moxifloxacin, Levofloxacin), p.Lys88Arg (rpsL - Streptomycin) |
| 8 | 647 | ECU_RDRio_49 | SRR40532546 | 4.3.4.2 | Susceptible | — | None detected |
| 9 | 1010 | ECU_RDRio_55 | SRR40532611 | 4.3.4.2 | Hr-TB | Isoniazid | p.Ser315Thr (katG - Isoniazid) |
| 10 | V 908 | ECU_RDRio_28 | SRR40532557, SRR40532556 | 4.3.4.1; 4.4.1.1 | MDR-TB | Rifampicin, Rifapentine, Isoniazid | p.Leu452Pro (rpoB - Rifampicin, Rifapentine), p.Ser315Thr (katG - Isoniazid) |
| 11 | MACH 11 | ECU_RDRio_21 | SRR40532560, SRR40532559 | 4.3.4.1 | Hr-TB | Isoniazida | p.Ser315Thr (katG - Isoniazid) |
| 13 | MACH 847 | ECU_RDRio_26 | SRR40532621 | 4.3.4.1 | MDR-TB | Rifampicin, Rifapentine, Isoniazid, Streptomycin, Ethionamide Prothionamide | p.Ser450Leu (rpoB - Rifampicin, Rifapentine), c.-777C>T (inhA - Isoniazid), c.114_256del (gid - Streptomycin), c.-777C>T (inhA - Ethionamide, Prothionamide) |
| 16 | CCA 874 | ECU_RDRio_27 | SRR40532620 | 4.3.4.1 | MDR-TB | Rifampicin, Rifapentine, Isoniazid, Streptomycin, Ethionamide Prothionamide | p.Ser450Leu (rpoB - Rifampicin, Rifapentine), c.-777C>T (inhA - Isoniazid), c.114_256del (gid - Streptomycin), c.-777C>T (inhA - Ethionamide, Prothionamide) |
| 17 | VAL 1331 (4) | ECU_RDRio_34 | SRR40532617 | 4.3.4.1 | MDR-TB | Rifampicin, Rifapentine, Isoniazid, Ethambutol | p.Asp435Val (rpoB - Rifampicin, Rifapentine), p.Ser315Thr (katG - Isoniazid), p.Met306Val (embB - Ethambutol) |
| 18 | VAL 1331 (5) | ECU_RDRio_36 | SRR40532615 | 4.3.4.1 | MDR-TB | Rifampicin, Rifapentine, Isoniazid, Ethambutol | p.Asp435Val (rpoB - Rifampicin, Rifapentine), p.Ser315Thr (katG - Isoniazid), p.Met306Val (embB - Ethambutol) |
| 19 | 1345 | ECU_RDRio_38 | SRR40532613 | 4.3.4.1 | Susceptible | — | None detected |
| 20 | 1252 | ECU_RDRio_42 | SRR40532612 | 4.3.4.1 | Susceptible | — | None detected |
| 21 | 1385 | ECU_RDRio_45 | SRR40532552 | 4.3.4.1 | Susceptible | — | None detected |
| 22 | 609 | ECU_RDRio_46 | SRR40532551 | 4.3.4.1 | Susceptible | — | None detected |
| 24 | 612 | ECU_RDRio_47 | SRR40532550 | 4.3.4.1 | Susceptible | — | None detected |
| 25 | 678 | ECU_RDRio_51 | SRR40532549 | 4.3.4.1 | Susceptible | — | None detected |
| 27 | 1064 | ECU_RDRio_53 | SRR40532638, SRR40532637 | 4.3.4.1 | Susceptible | — | None detected |
| 28 | 501 | ECU_RDRio_56 | SRR40532636, SRR40532635 | 4.3.4.1 | FQ-resistant, non-MDR/RR-TB | Moxifloxacin, Levofloxacin | p.Ala90Val (gyrA) and p.Asp94Asn (gyrA) — fluoroquinolone heteroresistance |
| 29 | 502 | ECU_RDRio_58 | SRR40532632, SRR40532631 | 4.3.4.1 | Susceptible | — | None detected |
| 30 | UIO 572 | ECU_RDRio_18 | SRR40532623 | 4.3.4 | MDR-TB | Rifampicin, Rifapentine, Isoniazid, Ethionamide, Prothionamide | p.Ser450Leu (rpoB - Rifampicin, Rifapentine), c.-154G>A (inhA - Isoniazid), c.-154G>A (inhA - Ethionamide, Prothionamide) |
| 31 | QUI 17 | ECU_RDRio_22 | SRR40532622 | 4.3.4 | MDR-TB | Rifampicin, Rifapentine, Isoniazid, Ethionamide, Prothionamide | p.Ser450Leu (rpoB - Rifampicin, Rifapentine), c.-154G>A (inhA - Isoniazid), c.-154G>A (inhA - Ethionamide, Prothionamide) |
| 32 | 1243 | ECU_RDRio_41 | SRR40532555, SRR40532554 | 4.3.3 | Susceptible | — | None detected |
| 33 | 630 | ECU_RDRio_48 | SRR40532577 | 4.1.1; 4.3.4.1 | Susceptible | — | None detected |
| 34 | VAL 1089 | ECU_RDRio_30 | SRR40532562, SRR40532561 | 4.1.1 | MDR-TB | Rifampicin, Rifapentine, Isoniazid | p.His445Asp - p.Ser450Leu (rpoB - Rifampicin, Rifapentine), p.Ser315Thr (katG - Isoniazid) |
| 35 | 681 | ECU_RDRio_52 | SRR40532566, SRR40532565 | 4.1.1 | Susceptible | — | None detected |

Drug-resistance profiles were predicted from WGS data using TBProfiler and are genotypic predictions, not phenotypically confirmed. n° and Isolate ID correspond to those in Table 1; SRA accessions correspond to NCBI BioProject PRJNA1524051 (two accessions indicate re-sequenced samples). Predictions were obtained with TBProfiler v6.6.5 (tbdb commit 0a909094) for all isolates, except the fine characterization of the fluoroquinolone-resistant isolate (Sample ID 501), which used TBProfiler v6.7.0 (who_v2+ database); this isolate showed gyrA heteroresistance (A90V ~25–45% and D94N ~60–70% of reads). Hr-TB, isoniazid-resistant, rifampicin-susceptible TB; MDR-TB, multidrug-resistant TB; Pre-XDR-TB, pre-extensively drug-resistant TB; FQ-resistant non-MDR/RR-TB, isolated fluoroquinolone resistance not meeting MDR/RR-TB criteria.
